# A droplet digital PCR assay to measure pilin antigenic variation frequency in *Neisseria gonorrhoeae*

**DOI:** 10.1128/msphere.00094-25

**Published:** 2025-04-23

**Authors:** Sarah J. Quillin, Di Luo, Aoife Gavagan, Arthur Prindle, H Steven Seifert

**Affiliations:** 1 Department of Microbiology-Immunology, Northwestern University Feinberg School of Medicine 12244, Chicago, Illinois, USA; 2 Department of Biochemistry and Molecular Genetics, Northwestern University Feinberg School of Medicine 12244, Chicago, Illinois, USA; 3 Center for Synthetic Biology, Northwestern University 3270, Evanston, Illinois, USA; 4 Department of Chemical and Biological Engineering, Northwestern University 3270, Evanston, Illinois, USA; 5 Chan Zuckerberg Biohub Chicago, Chicago, Illinois, USA; University of Kentucky College of Medicine, Lexington, Kentucky, USA

**Keywords:** *Neisseria gonorrhoeae*, pilus, antigenic variation, gene conversion, digital PCR

## Abstract

The strict human pathogen *Neisseria gonorrhoeae* (gonococcus [Gc]) infects an estimated 82 million individuals globally and is a World Health Organization-designated bacterial pathogen of public health importance due to escalating antimicrobial resistance. Gc vaccines have been hindered by Gc’s ability to evade immune surveillance in part by varying its major surface antigens like the type IV pilus. We developed a quick and precise method for measuring pilin antigenic variation (Av) frequency using droplet digital PCR (ddPCR) technology. Two fluorescent probes were designed to detect either the hypervariable tail region of silent pilin locus *pilS*3-copy 1 (S3C1) or a sequence conserved in all *pilE* variants (CYS2). The appropriate frequency of pilin antigenic variation is measured by the proportion of *pilE* amplicons carrying the recombinant S3C1 copy relative to the total pilE amplicons measured by CYS2. The ddPCR assay is specific for RecA-dependent pilin antigenic variation. The reduced frequency of pilin Av in strains lacking RecA-modulating recombination protein RecX and the DNA helicase RecQ confirms the ability of the assay to measure changes in pilin Av frequency. We used the ddPCR assay to determine that pilin Av frequency is altered by the colony densities on a solid medium. The ddPCR assay is an accurate, efficient way to measure Gc pilin Av frequency.

**IMPORTANCE:**

Gonorrhea is a sexually transmitted infectious disease of the human genital and nasopharyngeal mucosa caused by the host-restricted bacterium *Neisseria gonorrhoeae*. The rise of antibiotic-resistant gonorrhea is an urgent global threat to public health. Pilus antigenic variation is a gene conversion process that allows *N. gonorrhoeae* to evade host immune surveillance, and a mechanistic understanding of this process is crucial to understanding *N. gonorrhoeae* pathogenesis. This report shows that we can adopt a digital PCR methodology to quickly and accurately measure pilin antigenic variation.

## INTRODUCTION

*Neisseria gonorrhoeae* (the gonococcus or Gc) is the main causative agent of the sexually transmitted infection of gonorrhea. Gonorrhea has become a major global health concern due to rapidly escalating antimicrobial resistance, causing an estimated 82 million individual infections annually and leading the World Health Organization to designate it as a bacterial pathogen of public health importance (1). If untreated, gonorrhea infection can lead to pelvic inflammatory disease, ectopic pregnancy, disseminated infection, and neonatal blindness in infants (1).

Gc mainly colonizes the genital mucosa, but it can also colonize the ocular, nasopharyngeal, and anal mucosa (2–4). Gc infection does not generate immunity to infection, partially due to the ability of Gc to antigenically and phase vary several surface structures, including the type IV pilus, the opacity proteins, and the lipo-oligo-saccharide (5). The pilus is a major colonization factor involved in host cell adherence, twitching motility, transformation competence, and resistance to neutrophil killing (6–12).

**Fig 1:**
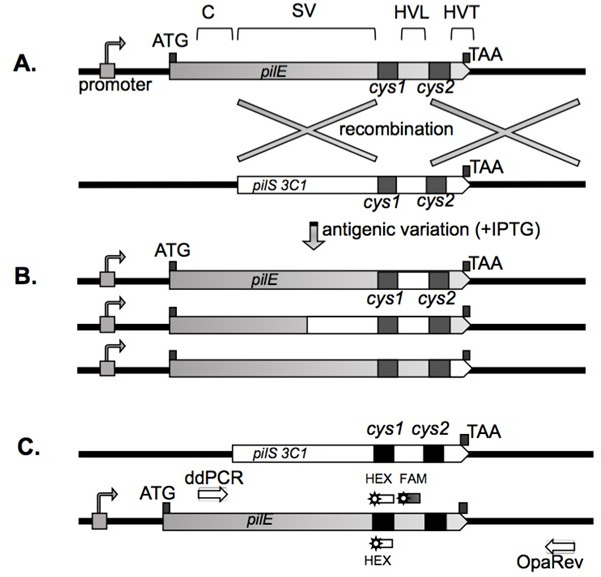
Diagram of the *pilE* locus, three possible recombination products of Av, and droplet digital PCR (ddPCR) assay reagent design. (**A**) Comparative diagram of genomic features present in the *pilE* locus and silent copy *pilS 3C1*. The *pilS* copies lack promoters and are not transcribed. The constant region “C” does not vary during Av, since it is not present in any *pilS* copies. The “SV” region is present in both *pilE* and the *pilS* silent copies with fewer sequence changes. The *cys1* and *cys2* regions are conserved between *pilE* and all *pilS* copies. The hypervariable loop “HV_L_” and hypervariable tail “HV_T_” are the most variable. (**B**) Diagram indicating three possible recombination products that may result during Av between *pilE* and *pilS 3*C1. The FA1090 *recA6* strain cannot undergo pilin Av unless isopropyl-β-d-thiogalactopyranoside (IPTG) is in the growth medium to induce RecA (+IPTG). (**C**) Diagram showing the location of the ddPCR and OpaRev amplification primers, the HEX-labeled cys1 probe, and the 6-carboxyfluorescein (FAM)-labeled *pilS 3C1* probe.

Pilin/pilus antigenic variation (Av) is mediated by a gene conversion process, where portions of 19 different unexpressed silent copies of the pilin gene (*pilS*) can transfer variant sequences into the sole pilin expression locus (*pilE*) (13–16). The pathogenic *Neisseria*, Gc and *Neisseria meningitidis,* both undergo pilin antigenic variation, whereas the commensal *Neisseria*, which occupy the same host-restricted mucosal niches, do not. Pilin Av recombination events are nonreciprocal, and recombination tracts are always bordered by regions of microhomology shared between each *pilS* copy and *pilE* (*17*) (Fig. 1A). The *pilS* silent copies lack the promoter, ribosome-binding site, and N-terminal pilin coding sequences, so the silent copies cannot produce pilin. Shared features between the *pilE* expressed locus and the *pilS* silent copies are the highly conserved *cys1* and *cys2* regions, the hypervariable loop HV_L_ located between *cys1* and *cys2*, and the hypervariable tail HV_T_ downstream of *cys2* (Fig. 1A). The semi-variable (SV) region immediately upstream of *cys1* is present in both *pilE* and *pilS* copies. The *cys1* and *cys2* regions encode conserved sequences that form disulfide cysteine bridges within the pilin and the pilus (18, 19). The SV region contains both conserved sequences and variable sequences. Following the HV_T_ and stop codon is the conserved SmaI/ClaI region downstream of *pilE* and downstream of the last *pilS* copy in each locus (20, 21). During a pilin Av event, as little as one bp sequence change may result, or an entire silent copy sequence may replace the starting *pilE* locus (22) (Fig. 1B). For reasons not fully understood, the frequency of recombination for the 19 *pilS* silent copies is not equal, and some copies undergo Av recombination events more frequently than others (22, 23). Pilus phase variation assays and next-generation sequencing assays have been used to measure surrogate frequencies of pilin antigenic variation (23–26).

Several trans-acting protein factors involved in DNA recombination and repair are involved in pilin Av (24, 27). Many trans-acting factors that affect Av also function in DNA repair and recombination and have well-studied orthologs in other bacterial species. In addition to its functioning in transformation and DNA repair during replication, the RecA recombinase is a major trans-acting factor required for Av through facilitating homologous recombination (28, 29). A *recA* mutant is Avd (28), and an isopropyl-β-d-thiogalactopyranoside (IPTG)-regulated *recA* gene has been used to control when pilin antigenic variation can occur since strains grown without IPTG are Avd, while strains grown with IPTG in the growth medium are Av proficient (29). Two RecA modulating factors, RdgC and RecX, are required for efficient pilin antigenic variation since loss-of-function mutants produce a reduced, intermediate antigenic variation (Avi) frequency (30–32). Members of the RecF-like pathway are also required for pilin Av (24, 27, 33), but a *recQ* mutant strain is Avi (27, 34–36). In addition to trans-acting factors that affect Av, few environmental conditions have been shown to affect Av, and more research is needed to understand the effects of the host mucosal environment and host immune response on Av. Iron limitation, a possible environmental result of host nutritional immunity, increases Av frequency, while other conditions like carbon source variation, aromatic amino acid availability, oxygen concentration, and temperature variation do not appear to affect Av (37, 38).

Many methods have been used to measure pilin antigenic variation. The most common method is to follow antigenic variation by scoring the number of nonpiliated progeny as a surrogate measure of pilin antigenic variation. This has often been done by counting P+ and P− colonies in a population, but a semi-quantitative assay observing pilus-dependent colony morphology changes (PDCMC) has also been used to measure antigenic variation frequencies (24). However, the PDCMC assay is very sensitive to changes in Gc growth and is inaccurate when a mutation or environmental conditions alter the growth rate. A reverse-transcriptase (RT) PCR assay and a Southern blot analysis have also been used to assay for pilin Av frequencies but are not quantitative (25). Sanger sequencing of individual *pilE* genes from several variants from a set number of founder colonies has been used to measure pilin Av (22). Currently, the most effective way to measure antigenic variation frequency is through long-read PacBio single-molecule, real-time sequencing of the *pilE* genes in a Gc population (26). Short-read sequencing methods can also be used, but presently, they cannot produce the sequences of individual *pilE* variants (39). A total of 454 pyrosequencing was used to measure antigenic variation frequencies (23), but this technique is no longer commercially available.

Droplet digital PCR (ddPCR) differs from conventional PCR because the reaction components are physically partitioned into discrete micro-reactions. Each droplet is amplified separately, allowing for absolute quantification, improved accuracy, and sensitivity (40). We developed a quick and reproducible method to measure pilin Av frequencies by ddPCR using a probe that targets a specific silent copy HV_L_ sequence that transfers to *pilE* at a high frequency (22) and a second probe that targets a conserved region in all *pilE* variants as an internal control. This assay is sensitive, reproducible, and recapitulates results determined with next-generation sequencing techniques.

## MATERIALS AND METHODS

### ddPCR primers and probes

All Taqman probes and primer parameters were obtained and analyzed using IDT OligoAnalyzer (IDT). Primers ddPCR (5′-CAAGTTTCCGAAGCCATCCT) and OpaRev (5′-GTTCCGGGCGGTGTTTC) were designed to amplify a 648 bp *pilE-*specific amplicon but not any *pilS* silent copy. A Taqman probe with a HEX fluorophore was designed to bind to the conserved *cys1* region present in all pilin gene copies both silent and expressed (5′-Hex–CGGTAAAATGGTTCTGCGGACAGCCGGTT). A Taqman probe containing a FAM fluorophore was designed to bind to the HV_L_ region of *pilS 3C1* (5′-FAM-TTTGACGTCGTCGGCTTTGGCGTCGT) but is not present in the 1-81-S2 *pilE* variant used in this study (Fig. 1C).

### Strains and growth conditions for gDNA preparation

Strain FA1090 1-82-S1 *recA6* (*37, 41–43*) was revived from −80°C freezer stocks and grown onto solid gonococcal base (GCB) medium (Difco) agar plus Kellogg supplements I and II (22.2  mM glucose, 0.68  mM glutamine, 0.45  mM cocarboxylase, and 1.23  mM Fe[NO_3_]_3_) for 18 hours. Ten colonies were streaked onto a GBC solid medium to grow confluent lawns for 22 hours in the presence and absence of 1 mM IPTG. Genomic DNA (gDNA) was prepared using the Qiagen QIAamp DNA Mini kit (51304). Between 300 and 1,000 ng of gDNA was treated with 1 µL (10 U/µL) restriction enzyme AseI overnight at 37°C (NEB R0526S), an enzyme that cuts *N. gonorrhoeae* DNA throughout the genome but does not cut within the *pilE* locus to produce gDNA template fragments that can be partitioned into the emulsified oil droplets required for ddPCR. AseI was inactivated at 65°C for 20 minutes, and gDNA was quantified by the Quant-iT High-Sensitivity dsDNA Assay Kit (Thermo Fisher Scientific Q33120).

### ddPCR reaction preparation

All gDNA dilutions and ddPCR master mixes were prepared and aliquoted under a sterile hood that had been thoroughly decontaminated to remove environmental DNA (44). When the hood was not in use, it was subjected to UV light to remove any residual environmental DNA contamination sources. gDNA template was diluted to 0.5 ng/µL in sterile water to add 1 ng total DNA in 2 µL of volume to each ddPCR. A ddPCR master mix was prepared containing 250 nM of HEX probe, 250 nM FAM probe, 900 nM ddPCR primer, 900 nM OpaRev primer, 2× ddPCR Supermix for Probes (No dUTP) (Biorad #1863024), and water. Each ddPCR sample was 20 µL. For multiple samples, 20 µL of the master mix was added to PCR strip tubes, and 2 µL of 0.5 ng/µL DNA was added to the reaction template. Tubes were vortexed heavily and spun down in a tabletop centrifuge, and 20 µL of the reaction was added to the sample wells of a DG8 cartridge (Biorad #1864008).

### ddPCR droplet generation

All subsequent pipetting of ddPCR samples was performed using aerosol-barrier (Rainin) pipet tips. ddPCR master mix-containing tubes were vortexed heavily and spun down in a tabletop centrifuge, and 20 µL of the reaction was added to the sample wells in a DG8 cartridge (Biorad #1864008). Seventy microliters of Droplet Generation Oil for Probes (Biorad #1863005) was added to the oil wells in the same DG8 cartridge. One DG8 gasket (Biorad # 1863009) was fitted over the cartridge in its holder. The cartridge and holder were placed into the QX200 droplet generator for generation. After droplets were generated, the gasket was removed, and 40 µL of droplets was slowly and carefully transferred into a 96-well ddPCR plate (Biorad #1864108). The ddPCR plate and droplets were sealed using Pierceable Foil Heat Seal (Biorad #1814040) that is adhered to the plate by the PX1 PCR Plate Sealer.

### PCR conditions and droplet fluorophore signal detection

The foil-sealed ddPCR plate was placed in a Biorad thermocycler, and the reaction proceeded at 95°C for 10 minutes, 94°C for 30 seconds, 56°C for 1 minute, 72°C for 1 minute (39×), and 98°C for 10 minutes. The annealing temperature and extension time were optimized for the specific probes used in this assay and for the relatively long 648 bp ddPCR amplicon, respectively. After the PCR, droplets were read for HEX and FAM signals in channels 1 and 2 of the QX200 Droplet Reader (Biorad) controlled by QuantaSoft software. All data were analyzed using QuantaSoft for HEX and FAM positive/negative signal threshold determination. Data were analyzed for quality control to ensure that all ddPCR runs contained over 11,000 total droplets, there were no droplets with FAM signal alone, and the no template controls (NTCs) did not contain significant FAM or HEX signal. The threshold for positive HEX and FAM droplets was manually set for each run, and the threshold was adjusted in comparison with the NTC controls to avoid false positives for each fluorophore.

### Isolation of a *pilE* 3C1 variant

We isolated a Gc strain that contains the silent copy 3C1 sequence in the *pilE* locus. We isolated this strain by inducing Av with IPTG in a *recA6* strain background, screening for 3C1 variants, and then removing IPTG to create a stable variant. Strain FA1090 *recA6* was used to generate colonies for Av analysis. First, the Gc strain FA1090 *recA6* was Sanger sequenced using *pilE*-flanking primers PilRBS (5′-TTTCCCCTTTCAATTAGGAG) and OpaRev to assure the presence of the unvaried *pilE* starting sequence. This unvaried strain was revived from the −80°C freezer storage and streaked on GCB agar overnight at 37°C with 5% CO_2_. The unvaried strain was then streaked on GCB + 1 mM IPTG agar overnight for 16 hours at 37°C with 5% CO_2_ during which time Av was induced. Single varied colonies were isolated and patched onto GCB agar in clonal populations without IPTG overnight for 16 hours at 37°C with 5% CO_2_ during which time Av did not occur, and the clonal colony populations maintained their variant sequences in the *pilE* locus without varying further. Patches measuring 1 cm × 1 cm on GCB agar were necessary to produce enough template for colony PCR, as single colonies did not produce enough template material.

With the knowledge that the 3C1 copy variants make up 1% of the total variant population (23), we sought to screen ~1,000 colonies via pooled colony PCR using a 3C1-specific primer that also bound the ddPCR assay FAM probe sequence. We designed the primer FAMProbeRight (5′-TTTGACGTCGTCGGCTTT) and performed pooled colony PCR with the PilRBS primer, which binds upstream of the *pilE* locus but not upstream of the silent copy loci. Pools of 10 patches were swabbed into 50 µL gonococcal base liquid (GCBL) with 25% glycerol and lysed for 5 minutes at 100°C, and 2 µL of this lysate was used as a template for PCR. The cryoprotected pools could then be frozen at −80°C for revival and plating for single colonies in the event of a positive band for 3C1 sequence in the *pilE* locus. An *Escherichia coli* vector containing a recombinant 3C1 *pilE* sequence was used as a positive control for these assays. After isolating a clonal population, its *pilE* 3C1 sequence was confirmed via Sanger sequencing.

### Spiking experiment to assess ddPCR Av frequency assay accuracy and limit of detection

For the spiking experiments, a predetermined ratio of genomically encoded 3C1 FA1090 *recA6* gDNA was mixed with an equivalent amount of unvaried FA1090 *recA6* 1-81-S2 starting sequence gDNA isolated from bacteria grown without IPTG. Genomic DNA isolation, quantification, and ddPCR assays were performed as previously stated. All sample proportions were mixed, processed, and analyzed on three different days.

### Colony density plating and quantification

Gc was seeded at different densities using one colony plucked with a sterile Whatman disc into 500 µL GCBL (liquid GCB+ with 0.042% sodium bicarbonate), serially diluted, and seeded at different densities onto GCB agar containing 1.0 mM IPTG. The plated dilutions were evenly dispersed using sterile glass beads and grown for 22 hours at 37°C. Colony densities were quantified by counting the total number of colonies on an entire plate and then dividing the total colonies per plate by the total plate area in square centimeters (area = π*r*^2^).

## RESULTS

### Development of the ddPCR assay

We developed a method based on ddPCR to measure Gc pilin antigenic variation frequencies. While the process of pilin antigenic variation involves the recombination of one or multiple *pilS* silent copies, this assay focuses solely on detecting the recombination of one high-frequency recombining *pilS* silent copy termed copy 3C1 (Fig. 1). Previous studies have shown there are nonrandom incorporations of silent donor copies into *pilE* during pilin Av when using the FA0190 1-81-S2 *recA6* strain, with specific silent copy sequences overrepresented in *pilE* variants (13, 22, 23, 26, 45). While overall pilin antigenic variation occurs at a 5%–11% frequency, the *pilS3* copy1 (S3C1) usually represents 11%–27% of overall events (22, 23, 26), allowing us to use recombination of the S3C1 HV_L_ sequence as a proxy for overall pilin antigenic variation.

We designed the PCR amplification probes (ddPCR and OpaRev) to specifically amplify the *pilE* locus without amplifying any silent copy to prevent nonspecific signals (23) (Fig. 1C). We also designed a HEX-labeled oligonucleotide probe that binds to the conserved *cys1* region of *pilE* and every *pilS* copy. We developed a FAM-labeled probe specific for the *pilS 3C1* HV_L_ region. During ddPCR, the ddPCR and OpaRev primers amplify the *pilE* gene, while the HEX signal measures all droplets containing a *pilE* gene. The FAM signal measures droplets with *pilS 3* C1 HV_L_ *pilE* variants.

### The ddPCR assay accurately measures S3C1 variants

**Fig 2:**
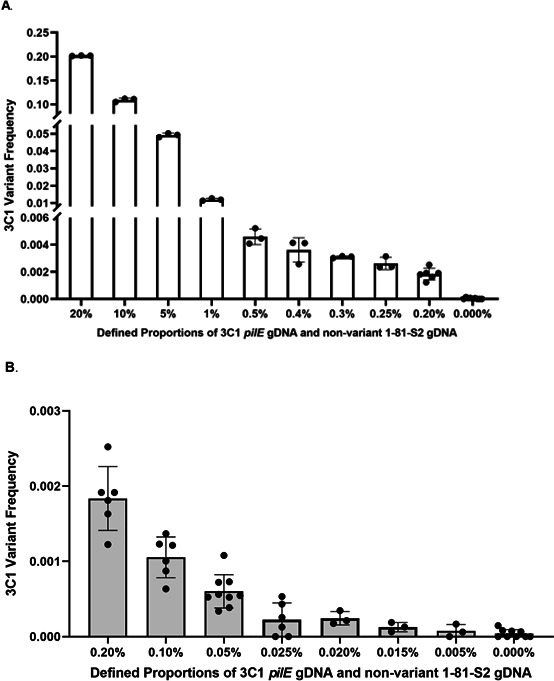
Reconstitution of pilin variation frequencies by spiking S3C1 *pilE* gDNA with non-variant 1-81-S2 *pilE* gDNA. To assess the accuracy, precision, and limit of detection of the ddPCR Av assay, we mixed defined proportions of chromosomal S3C1 *pilE* variant DNA and gDNA containing nonvariant 1-81-S2 *pilE* sequence (*x* axis). 3C1 variant frequency is represented as the proportion of FAM/HEX ratio (*y* axis). All solid, round data points for each separate proportion on the *x* axis represent a distinct biological replicate. (**A**) The assay can accurately measure the 3C1 variant frequency from 0.2% to 20% with a minimum of three biological replicates. (**B**) To reproducibly measure lower pilin Av, a minimum of six biological replicates is necessary.

To measure antigenic variation frequency by ddPCR, we utilized a strain where *recA*, required for antigenic variation to occur, is under the control of the *lac* regulatory system and thereby inducible with 1 mM of IPTG. This strain, called FA1090 *recA6*, has been thoroughly characterized for use in studies quantifying antigenic variation frequency and assures that the Gc begins agar plate growth with the same starting sequence, 1-81-S2. We screened for and isolated a naturally occurring 3C1 pilE variant using this strain. To assess the accuracy and precision of the ddPCR antigenic variation assay, we mixed defined proportions of chromosomal S3C1 *pilE* variant DNA and genomic DNA containing nonvariant 1-81-S2 *pilE* sequence (Fig. 2). Pre-defined input DNA ratios ranging from 20% 3C1 variant DNA to 0.20% 3C1 variant DNA resulted in precise, accurate output FAM/HEX 3C1 variant frequencies with three biological replicates (Fig. 2A). Below input DNA ratios of 0.20%, six biological replicates were required to maintain accuracy (Fig. 2B). Accuracy was lost below 0.05% 3C1 variant frequency with six biological replicates, revealing 0.05% 3C1 variant frequency as the level of detection for this assay.

### The ddPCR assay measures different frequencies of pilin Av

**Fig 3:**
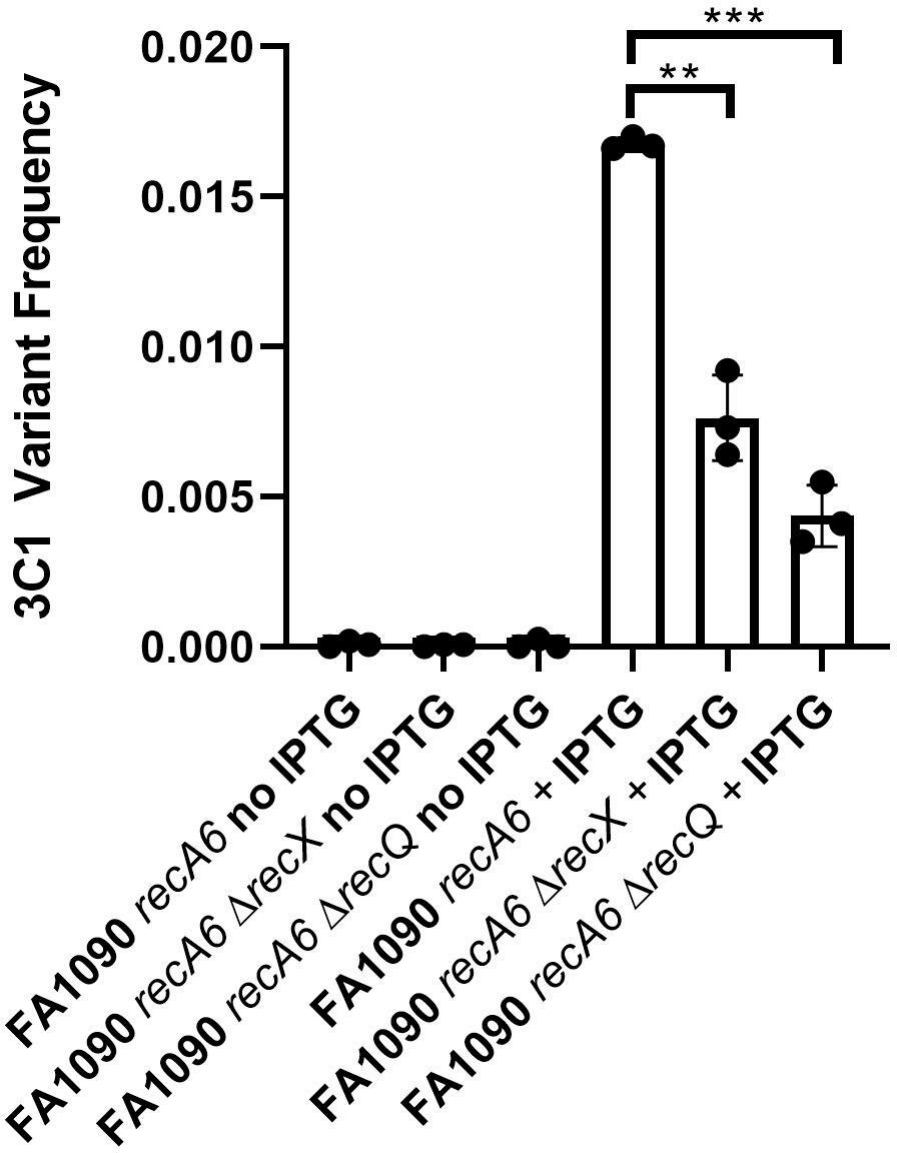
Testing whether the ddPCR assay can measure different pilin Av frequencies. The ddPCR assay to measure antigenic frequency was performed on gDNA harvested from three different FA1090 *recA6* strains in the presence and absence of 1 mM IPTG. The *x* axis denotes the strain description and whether 1 mM IPTG was present (+IPTG) or absent (no IPTG) in the solid agar medium during 22 hours of growth at 37°C. The FAM/HEX ratio indicating S3C1 variant frequency is on the *y* axis. All solid, round data points for each strain and condition on the *x* axis represent separate biological replicates performed on 3 different days. Black bars amidst the three biological replicates indicate the mean *y* value for each strain and one SD above and below the mean. * denotes *y* values that differ from one another in a statistically significant manner between different strains using Student’s unpaired *t* test (*P* < 0.05).

We grew FA1090 *recA6* 1-82-S1 for 22 hours of solid agar plate growth in the presence and absence of IPTG and isolated total genomic DNA. We conducted the ddPCR assay to determine the population S3C1 variant frequency. In the presence of IPTG, the average S3C1 variant frequency was 0.1% S3C1 per *pilE* (Fig. 3). This value is in close agreement with the S3C1 antigenic variation frequency in this strain measured by PacBio sequencing (26). The ddPCR assay did not detect any anomalous variation in the strain growth without IPTG (Fig. 3).

To test whether the ddPCR assay could measure lowered Av frequencies caused by trans-acting factors involved in DNA recombination, we assayed two mutant Gc strains previously shown to have reduced Av frequencies. The FA1090 *recA6 recX* strain contains an *erm* cassette in the open reading frame of *recX* (hereby referred to as Δ*recX*) and does not produce RecX protein. RecX has been shown to inhibit RecA in Gc by promoting rapid RecA filament disassembly (30, 46). A 454 pyrosequencing assay reported that the FA1090 1-81-S2 *recA6* Δ*recX* strain had an overall Av frequency of 3.44% (23). This same assay measured FA1090 *recA6* 1-82-S1 overall Av frequency as 10.6%–10.8% (23). The ddPCR assay measured the FA1090 *recA6* Δ*recX* strain mean 3C1 Av frequency value as 0.76% compared to 1.7% 3C1 Av frequency for the FA1090 *recA6* parental strain (Fig. 3). The overall Av frequency fold change reduction between the parental strain and Δ*recX* measured by 454 pyrosequencing is 3.1-fold, while the 3C1 Av frequency fold change reduction measured by ddPCR between the parental strain and Δ*recX* is 2.2-fold. While not identical, comparing these values shows that the ddPCR assay can measure reduced frequencies of pilin Av when comparing two strains or conditions.

In addition, we tested an FA1090 *recA6* Δ*recQ* mutant strain containing an *erm* cassette in the open reading frame of *recQ* that does not produce 3′-5′ DNA helicase RecQ (hereby referred to as Δ*recQ*) (34). A 454 pyrosequencing Av assay reported that FA1090 *recA6* Δ*recQ* has an overall antigenic variation frequency of 2.61% compared to an overall antigenic variation frequency of 10.6%–10.8% for the parental strain. The ddPCR Av assay showed an S3C1 variant frequency of 0.44%, compared to 1.7% for the FA1090 *recA6* parental strain (Fig. 3). The overall Av frequency fold change reduction between the parental strain and *ΔrecQ* measured by 454 pyrosequencing is 4.4-fold, while the 3C1 Av frequency fold change reduction measured by ddPCR between the parental strain and Δ*recQ* is 3.7-fold.

### Colony density affects antigenic variation frequency

**Fig 4:**
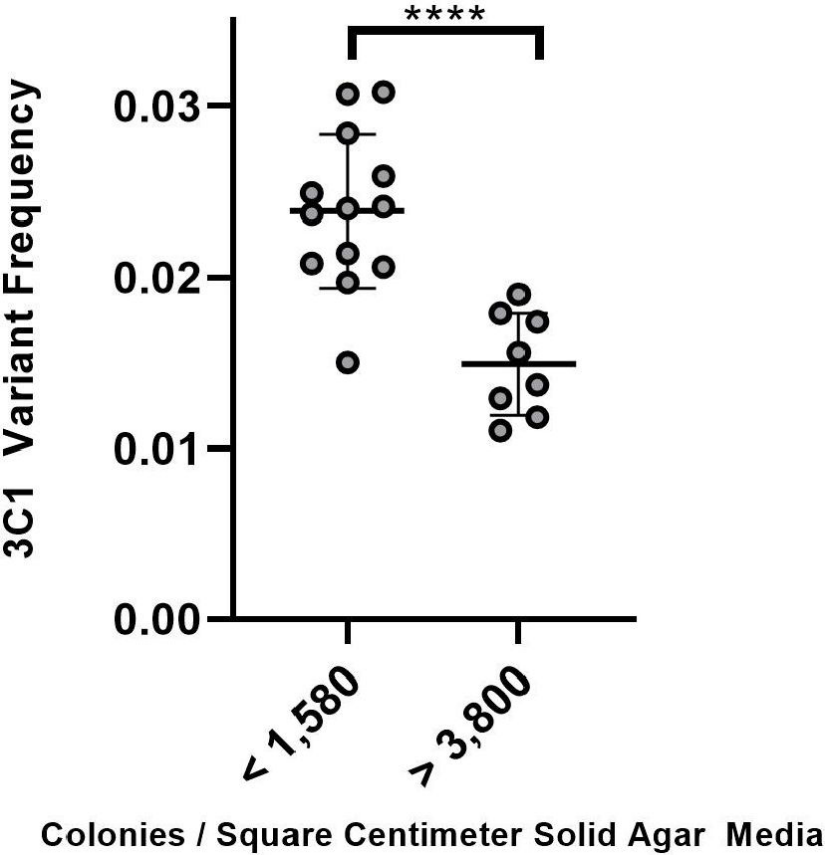
Colony density influences the pilin Av frequency. The ddPCR assay to measure Av frequency was performed on gDNA harvested from solid GCB agar plates seeded with a range of colony densities, then grown for 22 hours at 37°C. Colony densities on a solid agar medium are represented by colonies per square centimeter, as shown on the *x* axis and displayed in low- and high-density bins. FAM/HEX ratio indicating S3C1 variant frequency is shown on the *y* axis. Gray rounded data points represent individual solid agar plates from which gDNA was harvested on separate days. Black bars amidst the clustered samples indicate the mean *y* value for the two bins (<1,580 and >3,800 colonies/cm^2^) and one SD above and below the mean. * denotes *y* values that differ from one another in a statistically significant manner between two using Student’s unpaired *t* test (*P* < 0.05).

We used the ddPCR assay to determine whether colony density might affect Av frequency. FA1090 1-82-S1 *recA6* was seeded at a spectrum of colony densities on a solid agar medium. These variable colony densities were quantified by colonies per square centimeter of agar medium and represented as bins containing densities less than 1,580 cm^2^ or greater than 3,800 cm^2^ (Fig. 4 *x* axis). The pilin Av frequencies measured by the ddPCR assay showed that dense colonies showed lower Av frequencies when compared to colonies that were more spread out (Fig. 4).

## DISCUSSION

This report demonstrates that this ddPCR assay can reproducibly measure relative 3C1 pilin Av frequencies in pre-mixed ratios and ∆*recX* and ∆*recQ* mutant strains. The assay does not measure all antigenic variation events and, therefore, is only useful as a comparison between two samples, such as parent and mutant comparisons, or different environmental conditions, such as the low- and high-colony density conditions observed in Fig. 4. The higher 3C1 Av frequencies observed in low-density colonies are likely the result of more nutrient availability and subsequent growth under this condition, allowing for more DNA replication, recombination, and Av. As little is known about environmental conditions that affect Av, and past genetic screens have indicated unknown genetic factors contribute to Av, the ddPCR assay could be used to discover environmental and genetic factors affecting Av.

To increase the sensitivity of the assay, future experiments to optimize the current ddPCR assay to measure S3C1 variant frequency should focus on designing primers, probes, and PCR conditions that result in a shorter ddPCR amplicon and, thus, a shorter PCR extension time. The amplicon measured in this assay amplifies the expressed *pilE* locus, not any of the silent loci. In the current assay, the amplicon is considerably long for ddPCR at 648 bp, requiring a 90-second extension time. A smaller amplicon would lessen the extension time, considerably improving the efficiency of the assay. This method is currently limited to only detecting S3C1 variation and not overall antigenic variation. Despite this limitation, 3C1 Av frequency is still proper as a proxy for comparative trends in Av frequency, as exemplified by the trends in fold change reduction in Δ*recX* and Δ*recQ* mutant strains compared to the parental strain in Fig. 3. However, if different mutants affect the patterns of donor silent copy usage, this could affect the results of the ddPCR assay. No mutations have yet been shown to alter donor silent copy choice, but this does not eliminate this possibility.

Diversity generation systems like pilin Av exist in eukaryotic and prokaryotic organisms, often evolving in host-restricted organisms to evade host immune detection. In addition to the pathogenic *Neisseria*, bacterial Av occurs in Lyme spirochete *Borrelia burgdorferi* (47), gastrointestinal pathogen *Campylobacter fetus* (48), and rickettsial pathogen *Anaplasma marginale* (49, 50). Eukaryotic host-restricted malaria parasite *Plasmodium falciparum* and sleeping sickness parasite *Trypanosoma brucei* similarly undergo Av (51, 52). The evolution of Av is not exclusive to pathogens, however, as free-living ciliate *Paramecium* undergoes variation of its surface antigens (53), and the budding yeast *Saccharomyces cerevisiae* undergoes a type of Av to allow mating-type switching, varying its surface protein interactions with other yeast cells in the environment (54). Through specific primer and probe re-design, this assay may be adapted for other pathogenic and non-pathogenic prokaryotic and eukaryotic organisms that undergo gene switching, which may allow for forward genetic screening of unknown diversity generation determinants. In addition, this method could be expanded to study the frequency of gene rearrangement in human genes that undergo similar mechanisms to Av, such as T-cell antigen receptor gene rearrangement (55).

## Data Availability

The data underlying this article are available in Northwestern’s Arch data repository at https://doi.org/10.21985/n2-004h-es21.
